# Retinol‐Augmented PRF Versus PRF Alone in Periodontal Regeneration: A Randomized Clinical Trial

**DOI:** 10.1111/jre.13404

**Published:** 2025-05-21

**Authors:** Abdallah Hassan, Weam Elbattawy, Dalia Sanaa, Alaa Nawwar, Sameh Magdeldin, Maha Mokhtar, Taghreed Khaled Abdelmoneim, Karim Fawzy El‐Sayed

**Affiliations:** ^1^ Oral Medicine and Periodontology Department, Faculty of Dentistry Cairo University Cairo Egypt; ^2^ Periodontology Department, School of Dentistry Newgiza University Giza Egypt; ^3^ Oral and Maxillofacial Radiology Department, Faculty of Dentistry Cairo University Cairo Egypt; ^4^ Department of Oral and Maxillofacial Radiology, School of Dentistry Newgiza University Giza Egypt; ^5^ Proteomics and Metabolomics Research Program, Basic Research Unit, Research Department Children's Cancer Hospital Egypt Cairo Egypt; ^6^ School of Dental Medicine, Christian Albrechts University Clinic for Conservative Dentistry and Periodontology Kiel Germany; ^7^ Stem Cells and Tissue Engineering Unit, Faculty of Dentistry Cairo University Cairo Egypt

**Keywords:** minimally invasive surgery, periodontal, periodontitis, platelet‐rich fibrin, pocket, retinol, vitamin A

## Abstract

VitA/i‐PRF and i‐PRF alone improved clinical and radiographic outcomes of M‐MIST in periodontal intraosseous defects of patients with stage‐III grade B periodontitis. VitA/i‐PRF showed a minor, clinically non‐significant advantage in defect depth reduction and bone fill at 6 months, with effects diminishing by 9 months in patients with stage‐III grade B periodontitis.

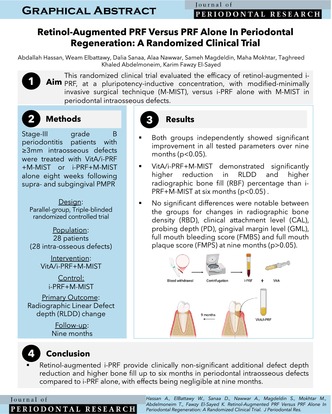

## 1 Introduction

Periodontitis associated with microbial dysbiosis could result in intraosseous defects. Periodontal therapy attempts to halt destructive disease processes via professional mechanical plaque removal (PMPR), surgical techniques, and adjunctive therapies. Minimally invasive approaches, as modified‐minimally invasive surgical technique (M‐MIST) [1], enhance flap/clot stability, wound healing, and blood supply in intraosseous defects. Injectable‐platelet‐rich fibrin (i‐PRF) [2] offers a sustained‐release vehicle of biomolecules for periodontal treatment [3]. Vitamin A, a fat‐soluble antioxidant with pleiotropic effects, affects cellular proliferation, growth/apoptosis, differentiation, and metabolism [4]. It enhances wound healing events, including angiogenesis, epithelial and fibroblast cellular turnover, as well as granulation tissue and collagen synthesis. Retinol, as ascorbic acid [5, 6], at specific concentration, possesses remarkable abilities to activate the Wnt/β‐catenin pathway and influence cellular epigenetics through demethylation of nuclear bases, inducing a pluripotent cellular state [7]. Thus, this trial evaluates the efficacy of sustained‐release retinol‐augmented i‐PRF, at a pluripotency‐inductive concentration, with M‐MIST, versus i‐PRF alone with M‐MIST in periodontal intraosseous defects.

## 2 Methods

**FIGURE 1:**
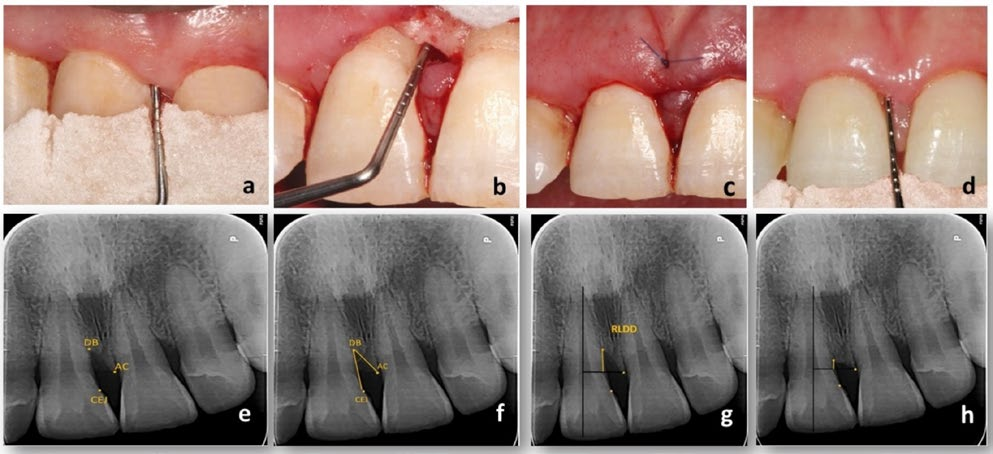
(a) Preoperative clinical photograph showing acrylic stent fabricated for standardization of the clinical measurements (b) Clinical photograph showing de‐granulated intra‐bony defect and confirming the depth ≥ 3 mm. (c) Clinical photograph showing the internal vertical mattress suture done using 6/0 Polypropylene (d) Clinical photograph showing probing depth after 9‐month follow‐up. (e) Periapical radiograph for the upper right central incisor showing three reference points: Alveolar crest (AC), defect base (DB) and cemento‐enamel junction (CEJ). (f) Periapical radiograph for the upper right central incisor showing the radiographic defect angle (RDA) of the mesial intraosseous defect formed between the three reference points. (g) Periapical radiograph for the upper right central incisor identifying reference lines: A vertical line corresponding to the long axis of the tooth and a horizontal perpendicular line passing through AC and identifying Radiographic linear defect depth (RLDD) of the pre‐treatment calculations. RLDD was measured as the depth of the intraosseous defect from the horizontal line passing through the AC to the DB. (h) Periapical radiograph for the upper right central incisor identifying RLDD of the post‐treatment calculations.

This 9‐month parallel‐group, triple‐blinded randomized controlled trial (RCT) (trial registration: NCT05285293, ethical approval: 19|9|21), where all participants signed informed consents, was conducted according to the ethical principles of the Helsinki Declaration revised in Fortaleza in 2013. Sample size was calculated for radiographic linear defect depth‐change (RLDD‐change) [8] with *α* = 0.05, *β* = 0.8, and effect size 1.221699, resulting in 12 participants/group (increased to 14 participants/group for 20% possible dropout, baseline characteristics, Table S1). Stage‐III grade B periodontitis (≥ 18 years), two‐and three‐walled intraosseous defect and probing depth (PD) ≥ 6 mm, clinical attachment loss (CAL) ≥ 4 mm, and intraosseous defect depth ≥ 3 mm following PMPR and full‐mouth bleeding or plaque scores ≤ 20% were only included (patient recruitment in Supporting Information). Suprabony, 1‐wall defects, grade II/III mobility, anticoagulant therapy, bleeding disorders, smokers, pregnancy/lactation, systemic illness known to influence the outcome of periodontal surgery as diabetes mellitus were excluded. Oral hygiene instructions, PMPR, and sub‐gingival instrumentation were completed and re‐evaluated after 6 to 8 weeks. Randomization into test (VitA/i‐PRF + M‐MIST) or control (i‐PRF + M‐MIST)‐groups with a 1:1 allocation ratio was concealed (KFE). Assignment of participants began after flap reflection, defect debridement, and intraosseous morphology confirmation. Inter‐observer agreement (Cronbach's alpha) of 0.945–1 was achieved for two blinded experienced investigators (WE) and (DS) recording all outcomes. RLDD‐change (primary outcome), CAL, PD, gingival marginal level (GML), full‐mouth bleeding score (FMBS), full‐mouth plaque scores (FMPS), radiographic bone fill (RBF) and radiographic bone density (RBD), all secondary outcomes, were assessed after 6 and 9 months with acrylic stents and bite blocks for standardization. Parallel‐angle technique (60kVp, 8 mA, 0.10s) was employed. Points were identified radiographically; alveolar crest (AC), defect base (DB) and CEJ to measure radiographic defect angle (RDA), RLDD, and RBF. RBD was assessed by calculating mean gray values for the region of interest (ROI, Figure 1).

Surgical intervention was performed via M‐MIST, through a horizontal internal bevel incision buccally at the base of the interdental papilla with minimal elevation without involving the neighboring papillae. A micro‐blade, mini‐curettes, magnification, and 6–0 monofilament polypropylene suturing material were used. In the test group, i‐PRF was prepared (700 rpm/3 min/RCF‐max = 60 g) with a centrifugation device (45° rotor angulation, SCILOGEX, CT, USA). Three milliliters of liquid i‐PRF was drawn into a sterile syringe and remained in a liquid state for 15–20 min before coagulation. Thirty‐three microliters of retinol (Vitamin A palmitate 1.7MIU/g in BHA/BHT, dsm‐firmenich, Kaiseraugst, Switzerland) was incorporated into i‐PRF (11 μL retinol for each 1 mL i‐PRF) to achieve a final concentration of 20μM^7^. Following gelation, VitA/i‐PRF was packed into the defect before suturing [9] (Figure 1). The same was performed in the control group but without vitamin incorporation. Ibuprofen 600 mg tid (Brufen, Kahira Pharma, Cairo, Egypt) was prescribed for 3 days, in addition to regular post‐surgical instructions and sutures removed after 14 days. Retinol in vitro release was examined using Ultra‐high performance liquid chromatography‐MS/MS (UHPLC/MS) (described in detail Appendix S1).

## 3 Results

This trial included 28 intraosseous defects in 28 patients (12 males/16 females). Two dropouts occurred during follow‐up with no adverse events. Significant improvement in RLDD was notable in each group after 6 and 9 months (*p* < 0.001) with no significant differences between groups (*p* > 0.05, Table S1). The VitA/i‐PRF + M‐MIST group showed significantly higher RLDD reduction and RBF than the i‐PRF + M‐MIST group after 6 months (*p* = 0.005), with no significant differences at 9 months (*p* > 0.05, Table 1). Although there was a significant increase within each group (*p* < 0.05), there was no inter‐group significant difference noted neither in RBD (*p* > 0.05, Table 1) nor for RBD changes at six and 9 months (*p* > 0.05, Tables S1 and 1). All clinical parameters (CAL, PD, GML, FMBS, and FMPS) showed significant improvement within each group from baseline to six and 9 months with no significant differences between the VitA/i‐PRF + M‐MIST and i‐PRF + M‐MIST groups at each timepoint (Table S1), in addition to no significant difference in CAL gain or PD reduction after 6 and 9 months (*p* > 0.05, Table 1). The stepwise linear regression model showed that age, gender, treatment (group), number of walls, PD, CAL, RDA, and RBD at baseline were not significant predictors of RLDD change. From day zero to day three, the retinol release showed a gradual increase from 0.09 to 0.18 μg/mL, demonstrating the highest release at day three (0.18 μg/mL), followed by a gradual decline in release over 7 days.

**TABLE 1:** Descriptive statistics and results of Mann–Whitney *U*‐test for comparison between changes in study outcomes in the two groups.

Outcome	Time	VitA/i‐PRF + M‐MIST (*n* = 14)		i‐PRF + M‐MIST (*n* = 12)		*p*	Effect size (d)
		Median (range)	Mean (SD)	Median (range)	Mean (SD)		
RLDD reduction	6 months	1.3 (0.67, 3.23)	1.46 (0.69)	0.73 (0.1, 1.63)	0.82 (0.42)	0.005*	1.299
	9 months	1.64 (0.78, 3.39)	1.65 (0.73)	1.45 (0.24, 1.76)	1.25 (0.51)	0.129	0.623
CAL gain	6 months	2 (0, 4)	2.36 (1.08)	2.5 (0, 4)	2.17 (1.19)	0.748	0.121
	9 months	2 (0, 5)	2.43 (1.34)	2 (0, 4)	2.2 (1.32)	0.814	0.091
PD reduction	6 months	3 (2, 4)	3 (0.55)	3 (1, 4)	2.75 (0.87)	0.442	0.265
	9 months	3 (2, 5)	3.21 (0.8)	3 (2, 4)	2.83 (0.72)	0.235	0.433
Change in GML	6 months	0.5 (0, 3)	0.64 (0.84)	0 (0, 2)	0.58 (0.79)	0.841	0.071
	9 months	0.5 (0, 3)	0.71 (0.91)	0 (0, 2)	0.58 (0.79)	0.732	0.121
Change in FMPS	6 months	1 (−1, 3)	0.71 (1.2)	1 (−1, 3)	0.69 (1.38)	0.980	0.131
	9 months	0.5 (−1, 3)	0.57 (1.22)	1 (−1, 3)	0.69 (1.38)	0.784	0.03
Change in FMBS	6 months	1 (−1, 2)	0.71 (0.83)	1 (−1, 2)	0.75 (0.87)	0.702	0.131
	9 months	1 (−1, 2)	0.57 (0.76)	1 (−1, 2)	0.58 (1.0)	0.912	0.04
Increase in bone density	6 months	5.6 (0.5, 15)	6.5 (5.4)	7.4 (0.4, 14.1)	7 (5.2)	0.758	0.121
	9 months	8.3 (1.6, 24.6)	9.4 (7.2)	12.4 (2.6, 34.5)	16.2 (10.2)	0.100	0.682
Bone fill %	6 months	31.7 (18.4, 60)	35.3 (14.1)	20.3 (3.1, 46.3)	23.9 (13.0)	0.031*	0.935
	9 months	40.7 (21.4, 63)	40 (14.0)	39 (7.4, 57.6)	36.5 (16.5)	0.681	0.162

* Significant at *p* ≤ 0.05.

## 4 Discussion

Besides supporting cellular activity and angiogenesis, i‐PRF acts as a slow‐releasing carrier when incorporated with a reparative/regeneration‐inductive biomolecule, with further augmentation of its effects in periodontal defects. As results of the in vitro release kinetics depicted, i‐PRF allowed for sustained release of retinol. Twenty micrometer retinol was incorporated into the i‐PRF, a concentration at which retinol influences cellular epigenetic memory, with epigenetic reprogramming driving de‐differentiation of adult cells into pluripotent ones and enhancing regenerative wound healing events [7]. Radiographic and clinical outcomes improved in both groups over time, yet the VitA/i‐PRF group demonstrated significantly higher RLDD reduction and bone fill percentage at 6 months. Retinoic acid, derived from retinol in the body, could have stimulated alkaline phosphatase activity, osteocalcin and osteonectin expression, and bone mineralization through BMP signaling and modulation of the Wnt/β‐catenin signaling pathway [10]. It was proposed that overexpression of cellular retinol binding protein‐1 promotes osteogenic differentiation through constraining RXRα‐induced β‐catenin degradation, preserving β‐catenin and pERK1/2, causing extended activation of ERKs, and enhanced osteogenesis [10]. Yet, the effects of the single sustained‐release retinol application over the first healing days could have faded after 9 months, as was reflected in the non‐significant differences between both groups.

Still, results should be carefully interpreted. Unique defect morphologies and strict inclusion of patients with stage‐III/grade B periodontitis may have limited the generalizability of findings. Second, observed clinical effects of in vivo retinol application may differ from earlier observed in vitro effects. Third, sample size calculation was based on a prior 6‐month trial, which might have contributed to the lack of significance at 9 months. Fourth, the use of 2D radiographic imaging for interpretation, an absence of patient‐reported outcomes, the relatively short follow‐up period, and the lack of histological analyses are further limitations. Fifth, comparing to a third arm using bone graft and barrier membrane could have given additional insights. Finally, an in vitro analysis of the osteogenic potential of amalgamated VitA/i‐PRF and the activated pathways would have strengthened the trial's findings.

In conclusion, i‐PRF can serve as a sustained‐release vehicle for biomolecules in periodontal therapy. Both interventions enhanced clinical and radiographic outcomes, yet retinol‐augmented i‐PRF seems to provide a clinically non‐significant additional defect depth reduction with higher bone fill up to 6 months in periodontal intraosseous defects compared with i‐PRF alone. These effects are negligible at 9 months. Fine‐tuning for retinol concentration and application frequency could be recommended in further clinical and histological studies with longer follow‐up and larger sample sizes.

## Author Contributions

All authors have made substantial contributions to the conception and the design of the trial. A.H., K.F.E.‐S., and W.E. conceived the idea and conducted the clinical trial. S.M., M.M., and T.K.A. conducted the in vitro experiment. K.F.E.‐S. led the writing. K.F.E.‐S., A.H., and W.E. conducted the data interpretation and led the writing. All authors revised the manuscript critically and gave final approval of the version to be published.

## Ethics Statement

The study protocols involving human participants adhered to the ethical standards set by the institutional and/or national research committee and were conducted in accordance with the principles outlined in the 1964 Helsinki Declaration and its subsequent revisions, or similar ethical standards. Both the research protocol and informed consent form were approved by the Ethics Committee of the Faculty of Dentistry, Cairo University, Egypt in September 2021 (IRB:19|9|21).

## Consent

All participants included in the study gave written informed consent before their participation.

## Supporting information


Appendix S1

Table S1

## References

[1] P. Cortellini and M. S. Tonetti , “Improved Wound Stability With a Modified Minimally Invasive Surgical Technique in the Regenerative Treatment of Isolated Interdental Intrabony Defects,” Journal of Clinical Periodontology 36, no. 2 (2009): 157–163. 19207892 10.1111/j.1600-051X.2008.01352.x

[2] F. Pullishery , M. Hussein Alattas , M. Roshdy Abdelrasoul , A. Fouad Hassan , D. Abdelhamid Ahmed Derbala , and S. Hashir , “Effectiveness of i‐PRF in Periodontal Regeneration – A Systematic Review and Meta‐Analysis,” Saudi Dental Journal 36, no. 2 (2024): 214–221. 38419983 10.1016/j.sdentj.2023.10.017 PMC10897594

[3] R. J. Miron , V. Moraschini , N. Estrin , et al., “Autogenous Platelet Concentrates for Treatment of Intrabony Defects‐A Systematic Review With Meta‐Analysis,” Periodontology 2000 97 (2024): 153–190. 39425513 10.1111/prd.12598 PMC11808470

[4] K. M. Fawzy El‐Sayed , R. Cosgarea , A. Sculean , and C. Doerfer , “Can Vitamins Improve Periodontal Wound Healing/Regeneration?,” Periodontology 2000 94 (2023): 539–602. 37592831 10.1111/prd.12513

[5] K. M. Fawzy El‐Sayed , A. Bittner , K. Schlicht , et al., “Ascorbic Acid/Retinol and/or Inflammatory Stimuli's Effect on Proliferation/Differentiation Properties and Transcriptomics of Gingival Stem/Progenitor Cells,” Cells 10, no. 12 (2021): 3310. 34943818 10.3390/cells10123310 PMC8699152

[6] K. M. Fawzy El‐Sayed , N. Nguyen , and C. E. Dorfer , “Ascorbic Acid, Inflammatory Cytokines (IL‐1beta/TNF‐Alpha/IFN‐Gamma), or Their Combination's Effect on Stemness, Proliferation, and Differentiation of Gingival Mesenchymal Stem/Progenitor Cells,” Stem Cells International 2020 (2020): 8897138. 32879629 10.1155/2020/8897138 PMC7448213

[7] K. M. Fawzy El‐Sayed , D. Hein , and C. E. Dörfer , “Retinol/Inflammation Affect Stemness and Differentiation Potential of Gingival Stem/Progenitor Cells via Wnt/β‐Catenin,” Journal of Periodontal Research 54, no. 4 (2019): 413–423. 30830694 10.1111/jre.12643

[8] M. T. Elbehwashy , M. M. Hosny , A. Elfana , A. Nawar , and K. Fawzy El‐Sayed , “Clinical and Radiographic Effects of Ascorbic Acid‐Augmented Platelet‐Rich Fibrin Versus Platelet‐Rich Fibrin Alone in Intra‐Osseous Defects of Stage‐III Periodontitis Patients: A Randomized Controlled Clinical Trial,” Clinical Oral Investigations 25, no. 11 (2021): 6309–6319. 33842996 10.1007/s00784-021-03929-1 PMC8531044

[9] C. C. Ho , D. Attia , and J. Liu , “Suturing Techniques,” in Practical Procedures in Implant Dentistry (Wiley, 2021), 155–162.

[10] Y. Liu , R. Zhang , X. Wang , et al., “All‐Trans Retinoic Acid Modulates Bone Morphogenic Protein 9‐Induced Osteogenesis and Adipogenesis of Preadipocytes Through BMP/Smad and Wnt/β‐Catenin Signaling Pathways,” International Journal of Biochemistry & Cell Biology 47 (2014): 47–56. 24300824 10.1016/j.biocel.2013.11.018

