# Supplementary material for: Retinol‐Augmented PRF Versus PRF Alone in Periodontal Regeneration: A Randomized Clinical Trial
Source: J Periodontal Res. 2025 May 21;60(8):847–9. doi: 10.1111/jre.13404 (PMC12476082; doi:10.1111/jre.13404)
Supplement: Supplementary file 1 — Appendix S1 [file JRE-60-847-s001.docx]

**Supplementary methods**

**Patient recruitment chart**

**
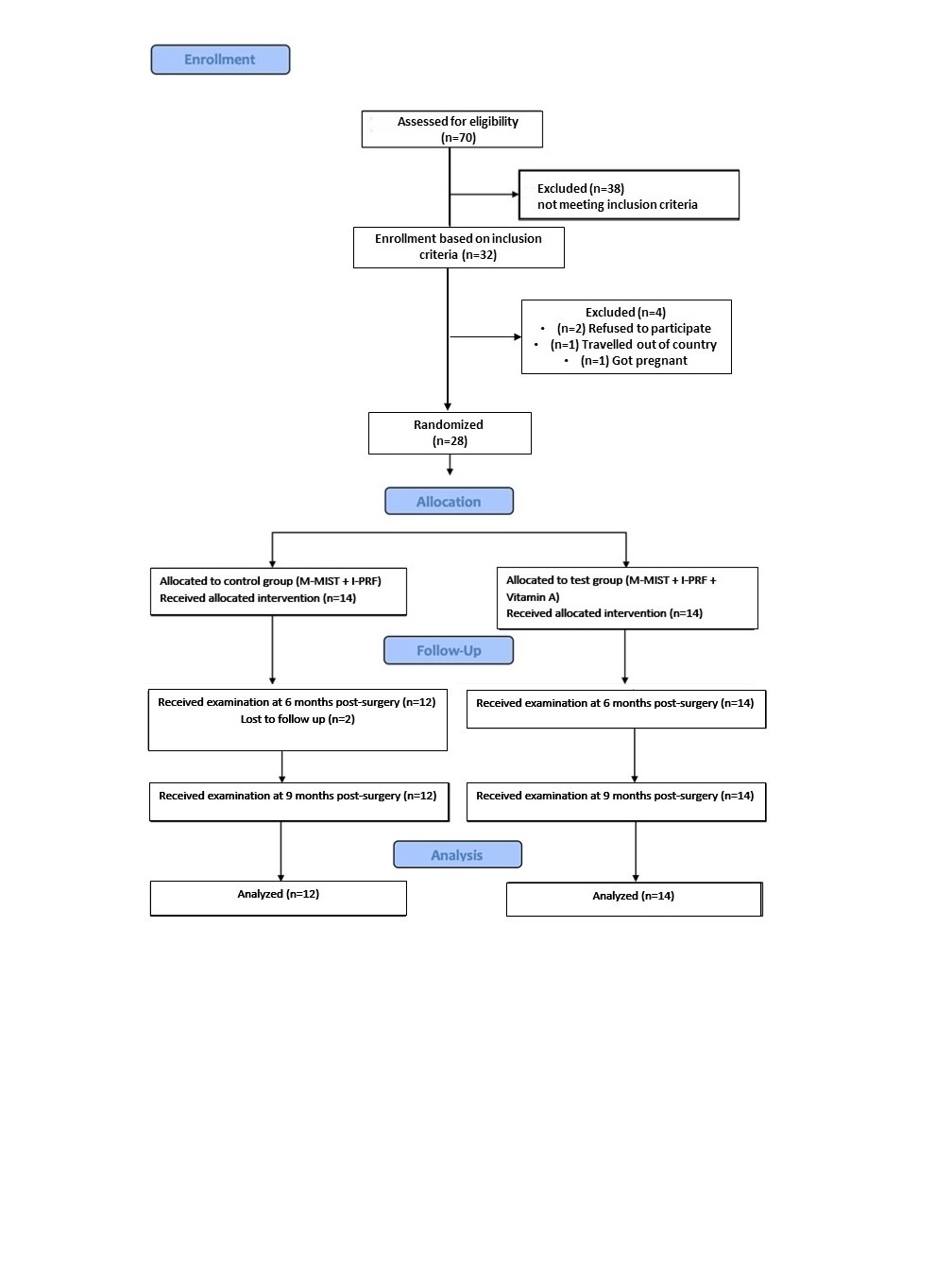
**

**In vitro release kinetics**

The in-vitro release of retinol was quantified using the modified analytical methods. The release was determined through immersing the i-PRF in 1 ml Phosphate Buffer Saline (PBS) and incubating at a temperature of 37°C. The release was measured at days zero, 1, 2, 3, 4, and 7 in the PBS, throughout removing the PBS at each time point and replacing it with new 1 ml of PBS. Then, the collected PBS at the different time points was subjected to quantification using Ultra-high performance liquid chromatography-MS/MS (UHPLC/MS). A standard curve of known concentrations of vitamin A was quantified throughout UHPLC/MS and used to determine the limit of quantification (LOQ) and limit of detection (LOD).

The ExionLC™ AC UHPLC analytical system was used with an Acquity XSelect HSS T3 analytical column (2.1 × 150 mm, 2.5 μm) at 40°C and a Triple TOF 5600+ mass spectrometer (AB SCIEX, Concord, Canada). The chromatographic separation was conducted by injecting 10 μL of each sample for 35 minutes using reverse phase gradient with a flow rate of 0.3 ml/min. The ionization was conducted on the positive mode, a combination of solution A (5 mM ammonium formate in 1% methanol (pH 3.0)) and solution B (100% Acetonitrile) was used as the mobile phase for the gradient elution. The gradient elution was employed at ratios of; 1 minutes of 95% solvent A, followed by, gradient of solvent B from 5 to 95% for 20 minutes, then 7 minutes holding period at 95% of solvent B, finally, 7 minutes of re-equilibration with 5% of solvent B. The positive ionization was conducted using a DuoSpray ion source (ESI+) mode for mass spectrometry. For the quantification of retinol in the tested samples, information-dependent acquisition with scanning mass ranges from 50 to 1000 Da in 30 milliseconds using Time-of-Flight Mass Spectrometry (TOF-MS). A fixed 50 Da transition window was used for each MS/MS acquisition, which lasted 50 milliseconds. The collision energy was set to 35 volts.

**Table S1:** Descriptive statistics of base line characteristics of patients in the two groups

| Patients’ data | VitA/i-PRF+M-MIST (n=14) | i-PRF+M-MIST (n=14) |
| --- | --- | --- |
| Gender [n, (%)] |  |  |
| Male | 8 (57.1%) | 4 (28.6%) |
| Female | 6 (42.9%) | 10 (71.4%) |
| Age [Mean, SD] | 42.4 (6.8) | 43.4 (7.8) |
| Arch [n, (%)] |  |  |
| Upper | 9 (64.3%) | 12 (85.7%) |
| Lower | 5 (35.7%) | 2 (14.3%) |
| Tooth [n, (%)] |  |  |
| Anterior teeth | 8 (57.1%) | 7 (50%) |
| Premolars | 4 (28.6%) | 7 (50%) |
| Molars | 2 (14.2%) | 0 (0%) |
| Number of walls [n, (%)] |  |  |
| Two walls | 3 (21.4%) | 7 (50%) |
| Three walls | 8 (57.1%) | 5 (35.7%) |
| Two and three walls | 3 (21.4%) | 2 (14.3%) |
| Defect angle [Mean, SD] | 40.7 (11.1) | 42 (8) |
