# Supplementary material for: Retinol‐Augmented PRF Versus PRF Alone in Periodontal Regeneration: A Randomized Clinical Trial
Source: J Periodontal Res. 2025 May 21;60(8):847–9. doi: 10.1111/jre.13404 (PMC12476082; doi:10.1111/jre.13404)
Supplement: Supplementary file 2 — Table S1 [file JRE-60-847-s002.docx]

**Table 1:** Descriptive statistics and results of repeated measures ANOVA test for comparison between RLDD measurements (mm), Bone Density, PD (mm), FMBS and FMPS in the two groups and the changes within each group as well as descriptive statistics and results of Mann-Whitney U test for comparison between CAL measurements (mm) and GML (mm) in the two groups and Friedman’s test for the changes within each group.

|  | VitA/i-PRF+M-MIST (n=14) | | i-PRF+M-MIST (n=12) | | *p*-value | | *Effect size*  *(Partial Eta squared)* | |  |
| --- | --- | --- | --- | --- | --- | --- | --- | --- | --- |
|  | Mean (SD) | | Mean (SD) | |  |  |  |  |  |
| **RLDD (mm)** |  | |  | |  | |  | |  |
| Base line | 4.08 (0.74)^A^ | | 3.55 (0.71)^A^ | | 0.073 | | 0.128 | |  |
| 6 months | 2.63 (0.66)^B^ | | 2.73 (0.84)^B^ | | 0.741 | | 0.005 | |  |
| 9 months | 2.43 (0.61)^C^ | | 2.29 (0.90)^C^ | | 0.650 | | 0.009 | |  |
| *p*-value | <0.001* | | <0.001* | |  | |  | |  |
| *Effect size*  *(Partial Eta squared)* | 0.800 | | 0.697 | |  | |  | |  |
| **Bone density** |  | |  | |  | |  | |  |
| Base line | 54.4 (10.3)^C^ | | 55.5 (18.1)^C^ | | 0.850 | | 0.002 | |  |
| 6 months | 60.9 (11.4)^B^ | | 62.5 (21.3)^B^ | | 0.806 | | 0.003 | |  |
| 9 months | 63.8 (11.1)^A^ | | 71.7 (19.7)^A^ | | 0.216 | | 0.063 | |  |
| *p*-value | 0.001* | | <0.001* | |  | |  | |  |
| *Effect size*  *(Partial Eta squared)* | 0.481 | | 0.633 | |  | |  | |  |
| **PD (mm)** |  | |  | |  | |  | |  |
| Base line | 6.71 (0.91)^A^ | | 6.5 (1.17)^A^ | | 0.605 | | 0.011 | |  |
| 6 months | 3.71 (0.83)^B^ | | 3.75 (0.97)^B^ | | 0.920 | | 0.0004 | |  |
| 9 months | 3.5 (0.83)^C^ | | 3.67 (0.89)^B^ | | 0.586 | | 0.013 | |  |
| *p*-value | <0.001* | | <0.001* | |  | |  | |  |
| *Effect size (Partial Eta squared)* | 0.916 | | 0.884 | |  | |  | |  |
| **FMBS** |  | |  | |  | |  | |  |
| Base line | 10.07 (1.49)^A^ | | 9.58 (1.31)^A^ | | 0.388 | | 0.031 | |  |
| 6 months | 9.36 (1.15)^B^ | | 8.83 (1.70)^B^ | | 0.360 | | 0.035 | |  |
| 9 months | 9.5 (1.40)^B^ | | 9 (1.60)^B^ | | 0.403 | | 0.029 | |  |
| *p*-value | 0.017* | | 0.021* | |  | |  | |  |
| *Effect size*  *(Partial Eta squared)* | 0.298 | | 0.285 | |  | |  | |  |
| **FMPS** |  | |  | |  | |  | |  |
| Base line | 10.5 (1.29)^A^ | | 10.5 (1.31) | | 1 | | 0 | |  |
| 6 months | 9.79 (1.67)^B^ | | 9.75 (1.76) | | 0.958 | | 0.0001 | |  |
| 9 months | 9.93 (1.82)^B^ | | 9.75 (1.76) | | 0.802 | | 0.003 | |  |
| *p*-value | 0.042* | | 0.173 | |  | |  | |  |
| *Effect size*  *(Partial Eta squared)* | 0.242 | | 0.142 | |  | |  | |  |
|  | |  | |  | | *p*-value | | *Effect size (d)* | |
|  |  | Median (Range) | | Median (Range) | |  |  |  |  |
| **CAL (mm)** | |  | |  | |  | |  | |
| Base line | | 6 (4, 10) ^A^ | | 6 (5, 9) ^A^ | | 0.354 | | 0.348 | |
| 6 months | | 4 (2, 8) ^B^ | | 3.5 (2, 6) ^B^ | | 0.833 | | 0.081 | |
| 9 months | | 3.5 (1, 8) ^B^ | | 3.5 (2, 6) ^B^ | | 0.833 | | 0.081 | |
| *p*-value | | <0.001* | | <0.001* | |  | |  | |
| *Effect size (w)* | | 0.864 | | 0.892 | |  | |  | |
| **GML (mm)** | |  | |  | |  | |  | |
| Base line | | 0 (-1, 2) ^A^ | | 1 (-1, 1) ^A^ | | 0.354 | | 0.348 | |
| 6 months | | 0 (-3, 2) ^B^ | | 0 (-1, 1) ^B^ | | 0.633 | | 0.182 | |
| 9 months | | 0 (-3, 2) ^B^ | | 0 (-1, 1) ^B^ | | 0.560 | | 0.223 | |
| *p*-value | | 0.001* | | 0.007* | |  | |  | |
| *Effect size (w)* | | 0.481 | | 0.417 | |  | |  | |

**: Significant at p≤0.05. Different superscripts in the same column indicate statistically significant change within group.*
